# Supplementary figures and images for: Commensal to pathogen switch in Streptococcus pneumoniae is influenced by a thermosensing master regulator
Source: PLoS Pathog. 2025 Sep 30;21(9):e1013545. doi: 10.1371/journal.ppat.1013545 (PMC12507249; doi:10.1371/journal.ppat.1013545)

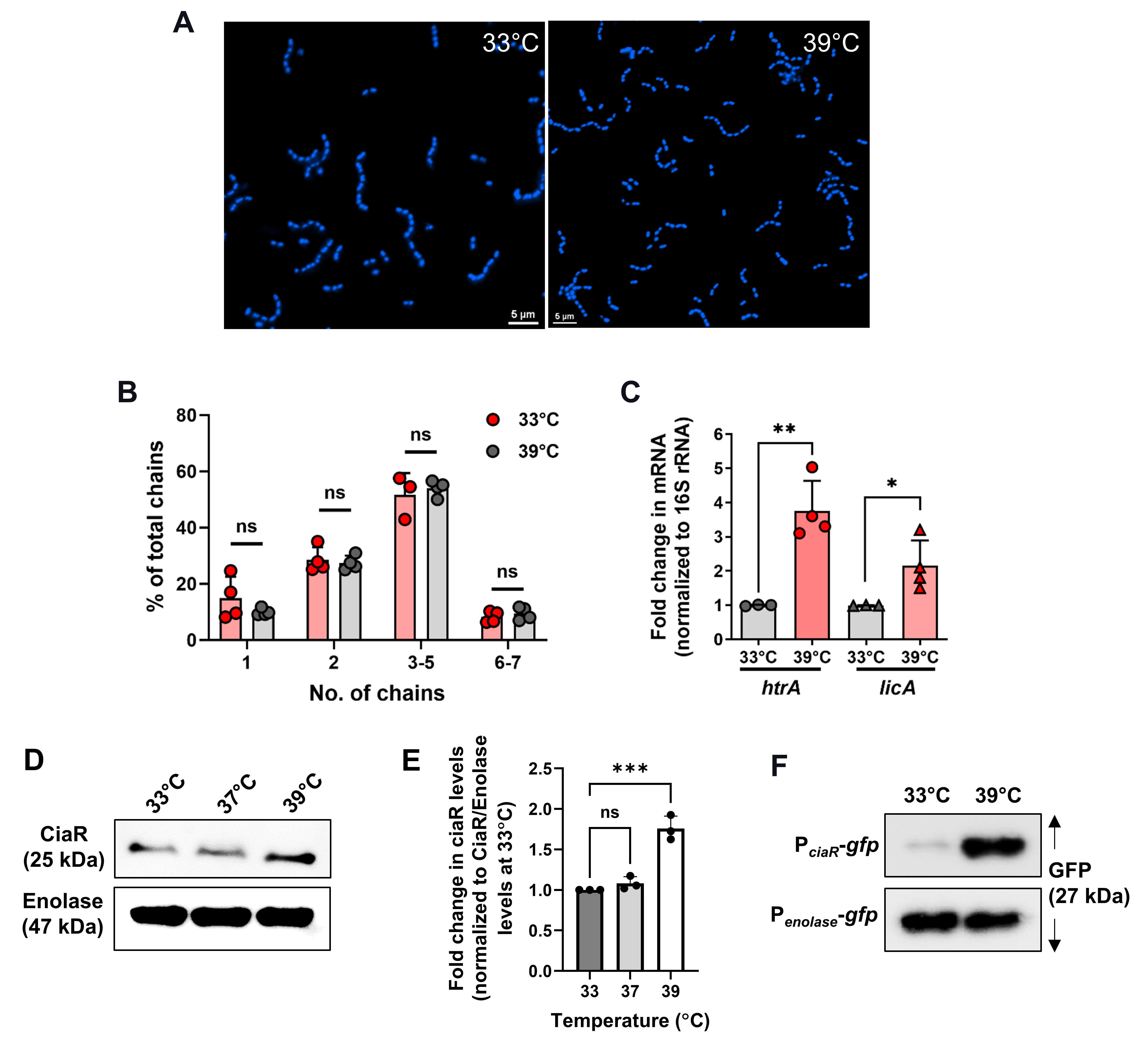

Supplement: S1 Fig — A. Representative Immunofluorescence image displaying SPN stained with Hoechst dye to quantify chain number. The temperature that they were exposed to before imaging is indicated in the left right corner. Scale bar, 5 μm. B. Bar graph displaying quantification bacterial chain number in WT SPN exposed to 33°C and 39°C. ImageJ (Fiji) software was used to quantify chain numbers and their frequency. C. qRT-PCR analysis of htrA and licA transcript levels in WT SPN exposed to different temperatures. 1 µg of total RNA isolated from SPN grown at different temperatures was reverse transcribed to synthesize cDNA and equal amount of cDNA from each test sample was used for analyzing Ct values using 2–∆∆Ct method. Transcript levels of each gene were normalized to 16S rRNA and expressed as fold change compared to 33°C. D. Immunoblot demonstrating CiaR levels in SPN exposed to 33°C, 37°C and 39°C. Enolase act as a loading control. E. Bar graph depicting fold change in CiaR levels at other temperatures compared to 33°C. CiaR levels at each temperature is normalized to corresponding Enolase levels. F. Immunoblot showcasing levels of GFP expressed under PciaR or Penolase promoters by after in vitro transcription, followed by in vitro translation at 33°C and 39°C. gfp mRNA was prepared first using DNA templates PciaR-gfp and Penolase-gfp and T3 RNA polymerase. Equal amounts of RNAs (~10 μg) were further translated using E. coli S30 Extract System at different temperatures. Statistical significance was assessed by two-tailed unpaired student’s t-test (B and C) or one-way ANOVA followed by Dunnett’s test (E). ns, non-significant; *P < 0.05; **P < 0.01; ***P < 0.005. Data are mean ± SEM of 3 independent biological replicates. (TIF) [file ppat.1013545.s001.tif]

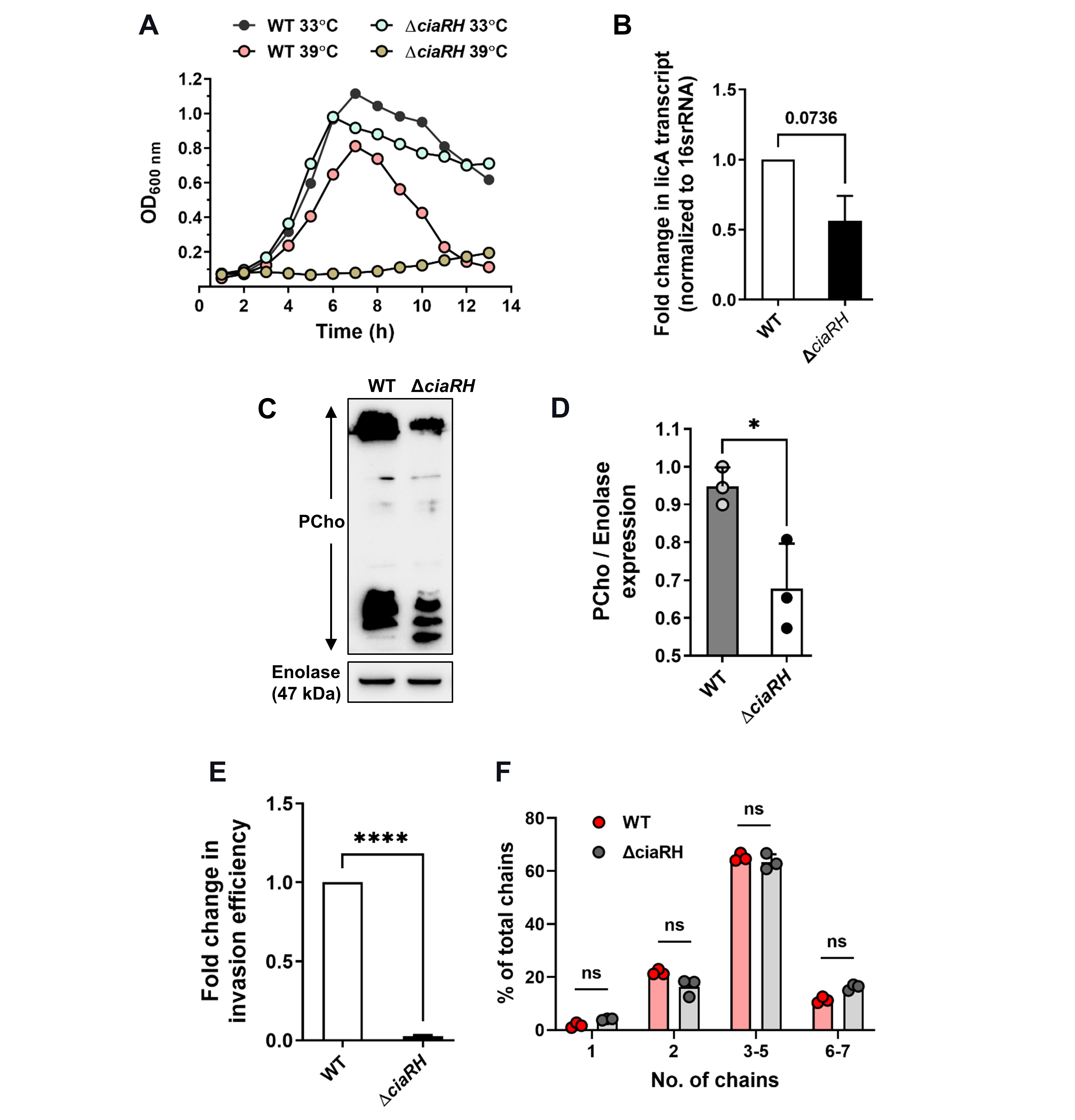

Supplement: S2 Fig — A. Growth curve comparing growths of WT and ΔciaRH SPN strain at 33°C and 39°C. B. Bar graph depicting abundance of licA transcript in WT and ΔciaRH SPN strains using qRT-PCR analysis. 1 μg of total RNA was reverse transcribed to synthesize cDNA and equal amount of cDNA from each test sample was used for analyzing Ct values using 2–∆∆Ct method. 16S rRNA transcript level was used to normalize the Ct values of each test transcripts and represented as fold change with respect to 33°C. C. Immunoblot demonstrating PCho levels in WT and ΔciaRH SPN. Enolase was used as a loading control. D. Densitometric quantification of ‘C’. E. Fold change in invasion efficiency of WT and ΔciaRH SPN in A549 cells at 37°C. Fold change was calculated as ratio of intracellular bacterial CFU of the ΔciaRH to that of WT SPN. F. Bar graph demonstrating the chain number of WT and ΔciaRH strain. ImageJ (Fiji) software was used to quantify chain numbers and their frequency. Statistical significance was assessed by two-tailed unpaired student’s t-test (D, E and F). *P < 0.05; **P < 0.01; ***P < 0.005. Data are mean ± SEM of 3 independent biological replicates. (TIF) [file ppat.1013545.s002.tif]

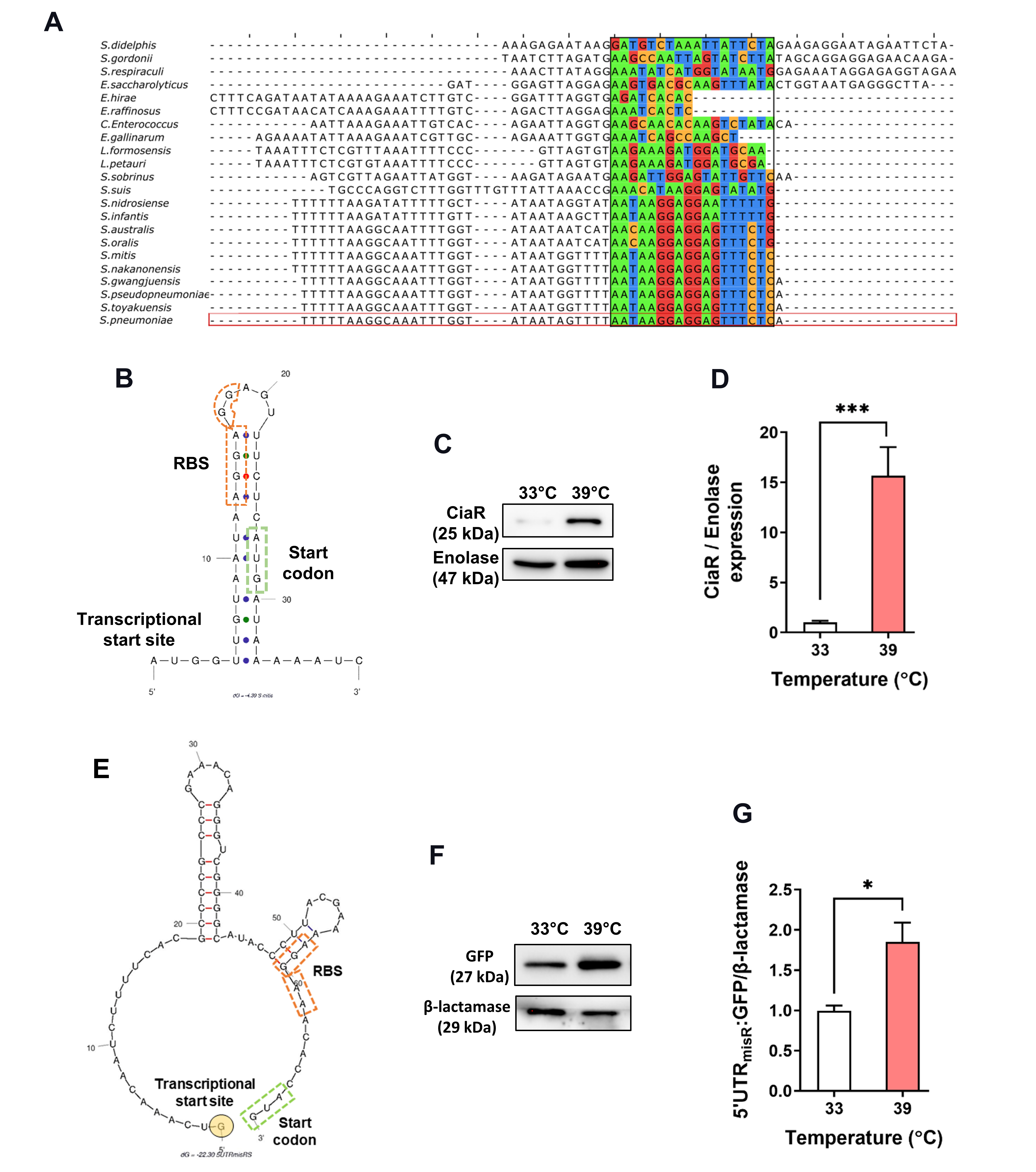

Supplement: S3 Fig — A. Sequence alignment of 5’-UTR ciaRH RNA-T like sequences in Streptococcus species and other bacteria. B. mFold predicted secondary structure of 5’-UTR of ciaRH of S. mitis marked with predicted Transcription Start Site (highlighted in yellow), Ribosome Binding Site (marked in orange) and Start codon (marked in green). C. Immunoblot demonstrating CiaR levels in S. mitis at 33°C and 39°C. Enolase served as loading control. D. Densitometric quantification of CiaR in ‘C’. E. mFold predicted secondary structure of 5’-UTR of misRS in Neisseria meningitidis marked with predicted Transcription Start Site (highlighted in yellow), Ribosome Binding Site (marked in orange) and Start codon (marked in green). F. Immunoblot of GFP expressed under the promoter of misRS operon. 20 μg protein was loaded for each sample and β-lactamase served as loading control. G. Graph representing densitometric quantification of ‘F’. Statistical significance was assessed by two-tailed unpaired student’s t-test (D and G). *P < 0.05; **P < 0.01; ***P < 0.005. Data are mean ± SD of 2 independent biological replicates. (TIF) [file ppat.1013545.s003.tif]

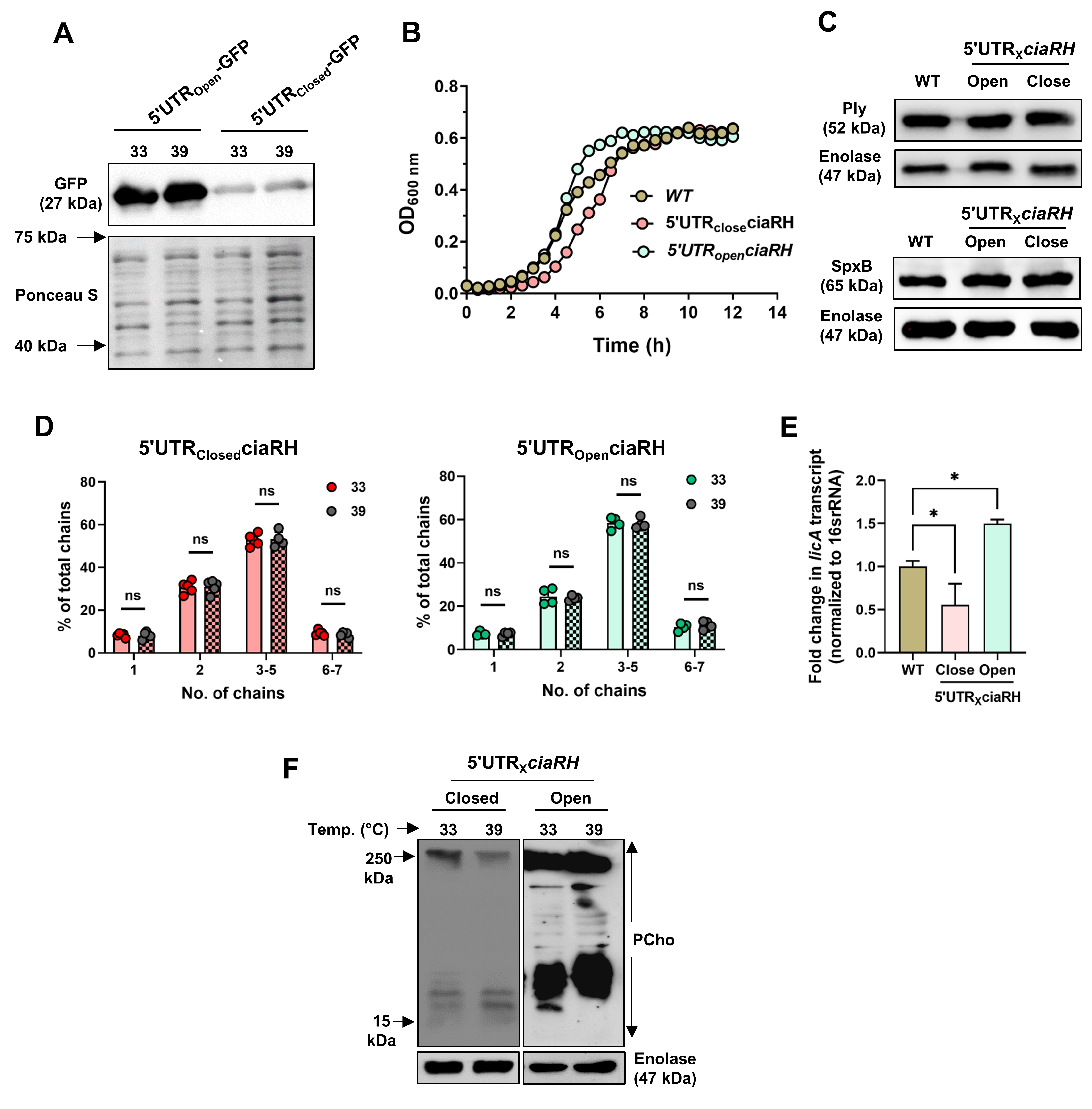

Supplement: S4 Fig — A. Immunoblot showcasing levels of GFP expressed at different temperatures from reporter strains carrying gfp gene under 5’-UTROpenciaRH and 5’-UTRClosedciaRH stretches. Ponceau S stained blot showcases equal protein loading. B. Plot displaying growth curve of WT and 5′-UTRClosed/Open ciaRH SPN strains at 37°C. C. Immunoblot depicting expression of SpxB and Pneumolysin in 5′-UTRClosed/Open ciaRH strains compared to WT SPN. 20 μ<delclass="ice−delice−cts−1"data−cid="84"data−userid="1"data−rolename="Author"data−username="abanerjee@iitb.ac.in"data−changedata=""data−time="1759247482763"data−last−change−time="1759247482763"data−action−time="1759313095142"data−action−user="collation@nkw.pub"data−action="Accepted"data−action−old−tag="del"data−action−hidden="Yes">g of total protein was loaded in each sample and enolase was used as loading control. D. Bar graph quantifying chain length and their frequency in mutated 5′-UTR ciaRH strains. Fiji software was used for marking and quantifying the pneumococcal cells and chains. E. qRT-PCR analysis of licA transcript level in 5′-UTRClosed/Open ciaRH and WT SPN strains. 1 μg of total bacterial RNA was used for reverse transcribed to cDNA and equal amount of cDNA from each test sample was used for analyzing Ct values. 16S rRNA transcript level was used to normalize the Ct values of each test transcripts using 2–∆∆Ct method. F. Representative immunoblot displaying PCho levels in WT and 5’-UTR ciaRH mutated strains of SPN. Enolase served as loading control. Statistical significance was assessed by two-tailed unpaired student’s t-test (C) and one-way ANOVA (Dunnett’s test) (D). *P < 0.05; **P < 0.01; ***P < 0.005. Data are mean ± SEM of 3 independent biological replicates. (TIF) [file ppat.1013545.s004.tif]

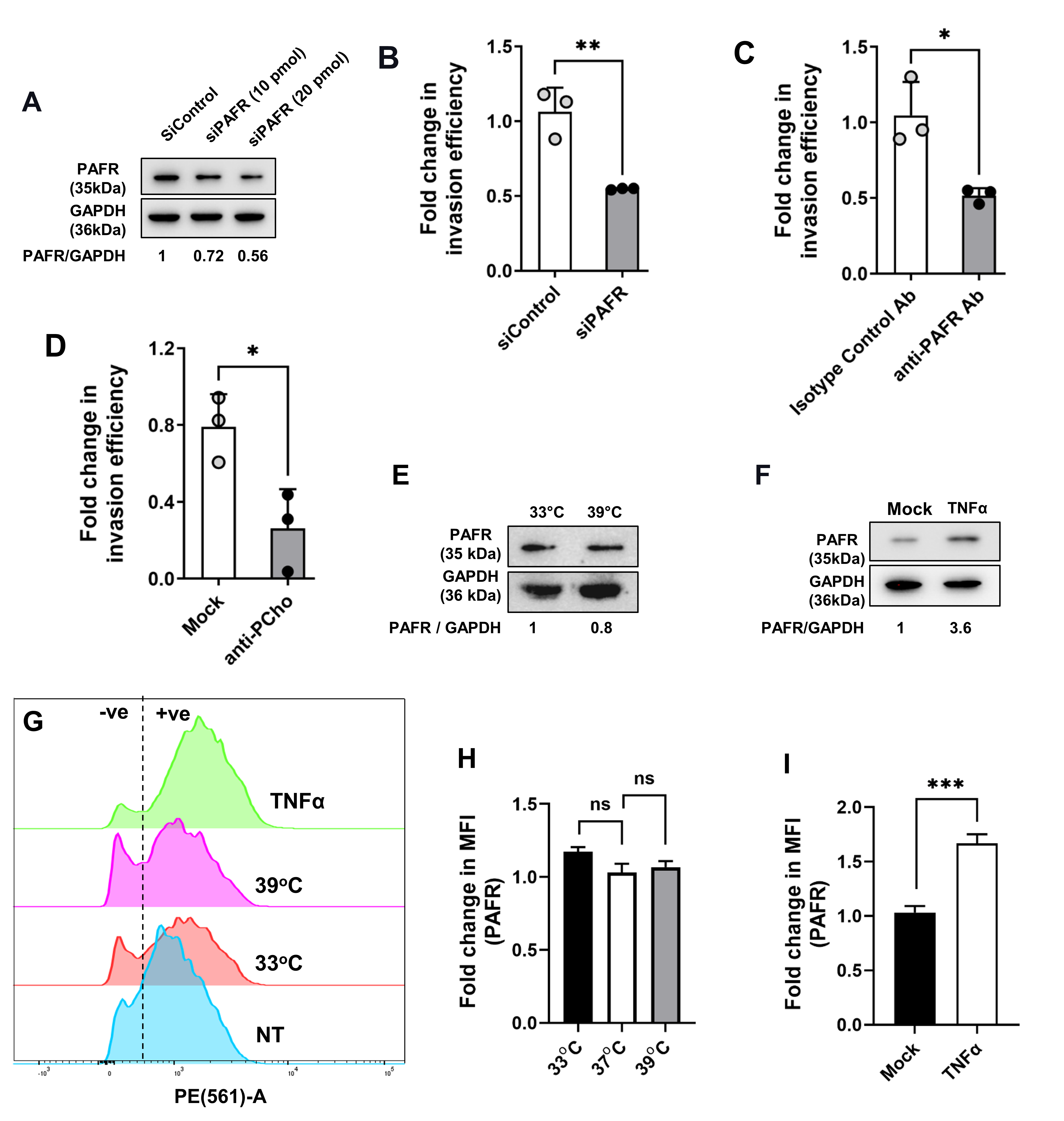

Supplement: S5 Fig — A. Immunoblot displaying PAFR levels in siControl and siPAFR treated A549 cells. Fold change in normalized PAFR levels with respect of GAPDH levels is mentioned below the blot. B-C. Graph depicting fold change in invasion efficiency of WT SPN in A549 cells treated with either siPAFR (20 pmol) for 24h (B); or blocked with anti-PAFR antibody (1:1000) for 2h prior to infection (C). Scrambled and isotype control antibody served as controls, respectively. Fold change was calculated as ratio of intracellular bacterial CFU of the test to that of control. E-F. Immunoblot of PAFR levels in A549 cells exposed to 33°C and 39°C (D) or treated with 30 ng/ml TNFα (E). Fold change in levels of PAFR in comparison to GAPDH in each test sample is denoted below the blot. G. Flow cytometry analysis demonstrating PAFR levels in A549 cells exposed to 33°C and 39°C for 6 h or following treatment with 30 ng/ml TNFα for 12 h. n ≥ 10000 cells were analyzed in 3 independent expts. and histogram reflects results obtained of one single expt. H. Graph depicting fold change in MFI (Mean Fluorescence Intensity) in PAFR signal in A549 cells exposed to 33, 37 and 39°C (H) or treated with TNFα (I) as acquired by flow cytometry. Fold change was calculated with respect to A549 cells grown at 37°C (unexposed) in H or mock treated in I. Statistical significance was assessed by two-tailed unpaired student’s t-test (B, C, D and I) and one-way ANOVA (Dunnett’s test) (H). no non-significant; *P < 0.05; **P < 0.01; ***P < 0.005. Data are mean ± SD of 3 independent biological replicates. (TIF) [file ppat.1013545.s005.tif]

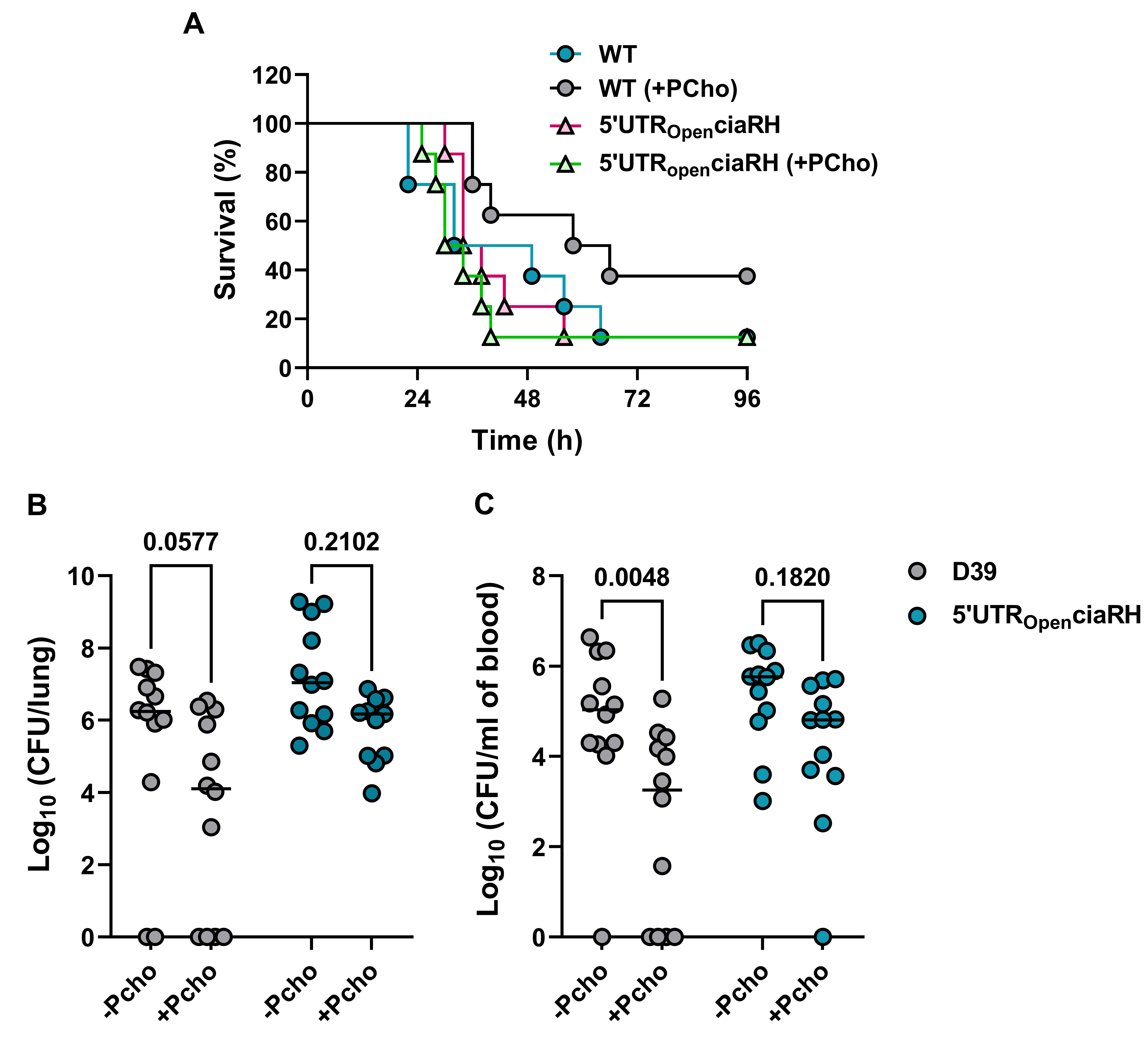

Supplement: S6 Fig — A. Mice were administered 2 mg PCho intranasally and two hours later were infected intranasally with 2x10^6^ CFU and monitored for signs of disease over 96 h. Mice were culled when they reached pre-determined disease severity endpoints. n = 10, *p < 0.05 in Kaplan-Meier survival analysis. B-C. Bacterial burden in lungs (B) and blood (C) at 24 h post-infection. Statistical significance was achieved by Mann-Whitney test and mentioned in each graph. (TIF) [file ppat.1013545.s006.tif]

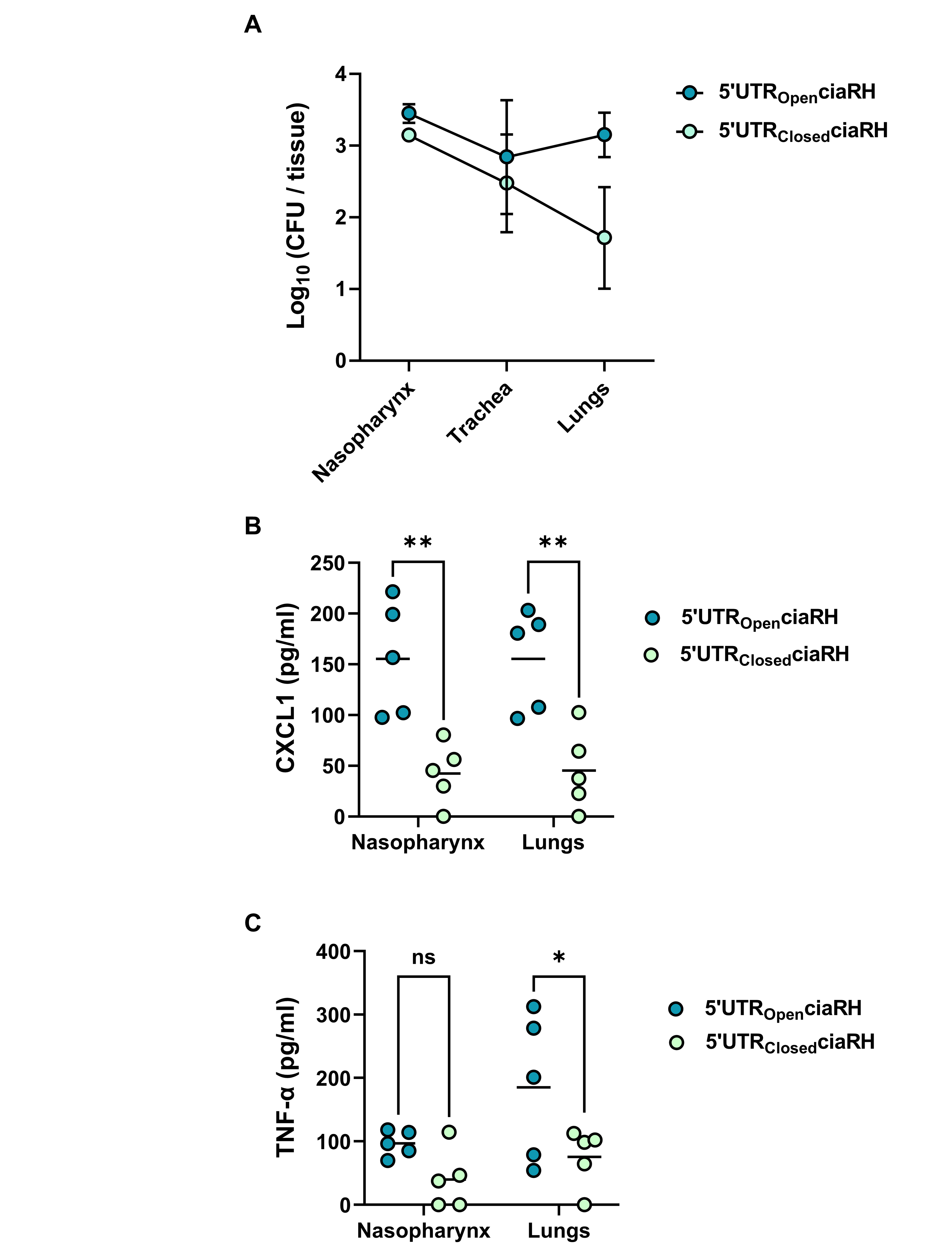

Supplement: S7 Fig — A. Mice were infected intranasally with 1x10^5^ colony forming units (CFU) and tissues were collected at three days post-infection. CFU in tissues was quantified by serial dilution onto gentamicin blood agar. B-C. CXCL1/KC (B) and TNF-α (C) were quantified in tissue homogenates by ELISA. ns = not significant, * = P < 0.05 and ** = P < 0.01 in two-way ANOVA analysis with Sidak’s multiple comparison testing. (TIF) [file ppat.1013545.s007.tif]
