# Supplementary material for: Commensal to pathogen switch in Streptococcus pneumoniae is influenced by a thermosensing master regulator
Source: PLoS Pathog. 2025 Sep 30;21(9):e1013545. doi: 10.1371/journal.ppat.1013545 (PMC12507249; doi:10.1371/journal.ppat.1013545)
Supplement: S1 Table — (DOCX) [file ppat.1013545.s008.docx]

**S1 Table. List of strains**

| **Strain** | **Source** |
| --- | --- |
| *Streptococcus pneumoniae* (SPN) strain D39 (Serotype 2, encapsulated) | Prof. E. Tuomanen (St. Jude Children's Hospital, USA) |
| D39Δ*ciaRH* | This study |
| D39Δ*ciaRH*: 5'UTR_closed_ *ciaRH* | This study |
| D39Δ*ciaRH*: 5'UTR_open_ *ciaRH* | This study |
