# Supplementary material for: Commensal to pathogen switch in Streptococcus pneumoniae is influenced by a thermosensing master regulator
Source: PLoS Pathog. 2025 Sep 30;21(9):e1013545. doi: 10.1371/journal.ppat.1013545 (PMC12507249; doi:10.1371/journal.ppat.1013545)
Supplement: S2 Table — (DOCX) [file ppat.1013545.s009.docx]

**S2 Table. List of Plasmids**

| **Plasmid** | **Details** | **Purpose** | **Source** |
| --- | --- | --- | --- |
| pAB309 | pBSK+promoterless GFP (without AUG) | As parent plasmid for reporter strains | This study |
| pAB368 | pAB178-CiaR | For creating WT ciaRH up down for genome integration of mutations | This study |
| pAB372 | pAB309+Penolase | Enolase reporter strain | This study |
| pAB376 | pAB368+5'UTR_open_ciaRH | For opening of 5'UTR of ciaRH | This study |
| pAB377 | pAB368+5'UTR_close_ciaRH | For closing of 5'UTR of ciaRH | This study |
| pAB382 | pBSK+CiaR-His6 (with its own promoter) | For checking the level of CiaR in *E.coli* | This study |
| pAB390 | pAB309+PmisR | MisR reporter strain | This study |
| pAB414 | pAB309+PciaRH | CiaRH reporter strain | This study |
| pAB1202 | pAB309+5'UTR_open_ciaRH | 5'UTR_open_ciaRH reporter strain | This study |
| pAB1203 | pAB309+5'UTR_close_ciaRH | 5'UTR_close_ciaRH reporter strain | This study |
