# Supplementary material for: Commensal to pathogen switch in Streptococcus pneumoniae is influenced by a thermosensing master regulator
Source: PLoS Pathog. 2025 Sep 30;21(9):e1013545. doi: 10.1371/journal.ppat.1013545 (PMC12507249; doi:10.1371/journal.ppat.1013545)
Supplement: S3 Table — (DOCX) [file ppat.1013545.s010.docx]

**S3 Table. List of Primers**

| **Primer name** | **Sequence (5'-3')** |
| --- | --- |
| GFP-F-BamHI | ATTTAAGGATCCAAAGGAGAAGAGCTGTTCACA |
| GFP-R-XhoI | GTTAGTCTCGAGTTACTTATAAAGCTCATCCAT |
| PciaR-F | GTAGGCTTCCTAATACGACTCACTATAGGGAATAAGGAGGAGTTTCTCATG |
| PciaR-R | GTTAGTCTCGAGTTACTTATAAAGCTCATCCAT |
| Peno-F | CTGACTGACGGATCCCATTTTTTACTCTCCTTATGAG |
| Peno-R | ACCGCGGTGGCGGCCGCTCTAGAGAGCTTTTTCAAGTA |
| open ciaR-F | GAATATAATGATAAAAATCTTATTGGTTGAGG |
| open ciaR-R | TCCTCCTTATTAAAACTATTATACCAAATTTG |
| closed ciaR-F | AACAAGGAGGTTCCCCCCATGATAAAAATCTTATTGGTTGAGG |
| closed ciaR-R | AAAACTATTATACCAAATTTGCCTTAAAAAAAAC |
| ciaR-F-pbsk-XhoI | TTGCATGCCTCGAGATAAGCCTAAAATAAAAAGAAAACTCAGCTATCTCATGTAA |
| ciaR-R-pbsk-His6-EcorI | ATCGATCGAGAATTCTTAGTGGTGATGGTGATGATGCTGAACATCTTTTAAAAGA |
| PmisR-F | GCTCTTCTCCTTTGGATCCCATGGTGTTTCCTTTTCGTAAG |
| PmisR-R | GCGGTGGCGGCCGCTCTAGACATGCAAGACATTGCAAAAA |
| 16SrRNA-F | AACCAAGTAACTTTGAAAGAAGAC |
| 16SrRNA-R | AAATTTAGAATCGTGGAATTTTT |
| Spec-R-BamHI | ATCCGGATCCAATCTGATTACCAATTAGAATG |
| specF2-BamHI | CCGCGGATCCCATATATAATCTAGAATAAAATTAAC |
| LicA-F-RT | CGATTTGGTGCCTGAAAACT |
| LicA-R-RT | accggtgtttggtcactctc |
| CiaR-F-RT | GATGGAGAAGAAGGTC |
| CiaR-R-RT | GTCATAATCAGAACTGG |
| HtrA-F-RT | GTTTCGCAATTCCTGCAAAT |
| HtrA-R-RT | TGGTGTAGTTGTTCGTTCGG |
| pciaRopen-SacI-FP | CTGACAGAGCTCGAATTCATAAGCCTAAAATAAAAAG |
| pciaRopen-ATG-BamHI-RP | CAGTCAGGATCCCATTATATTCTCCTCCTTATT |
| pciaRclosed-SacI-FP | CAGTCAGAGCTCCATAAGCCTAAAATAAAAAGAAAAC |
| pciaRclosed-ATG-BamHI-RP | CAGTCAGGATCCCATGGGGGGAACCTCCTTGTT |
