# Supplementary material for: Commensal to pathogen switch in Streptococcus pneumoniae is influenced by a thermosensing master regulator
Source: PLoS Pathog. 2025 Sep 30;21(9):e1013545. doi: 10.1371/journal.ppat.1013545 (PMC12507249; doi:10.1371/journal.ppat.1013545)
Supplement: Attachment — Submitted filename: Response to Reviewers_PPATHOGENS-D-25-01275.docx [file ppat.1013545.s013.docx]

**Response to Reviewers' Comments (PPATHOGENS-D-25-01275):**

**Part I - Summary**

**Reviewer #1:**

Apte and colleagues present a compelling story on a thermosensory RNA element located upstream of the ciaR gene, which encodes a master regulator in Streptococcus pneumoniae. Their findings demonstrate that elevated temperatures lead to increased CiaR levels. This induces the production of the surface adhesin phosphorylcholine (Pcho), which promotes host invasion and enhances virulence. In an extension of previous research on temperature-dependent capsule biosynthesis, this work uncovers another link between temperature change and bacterial surface modification.

The manuscript contains a substantial body of rigorously conducted work, well within the scope of PLoS Pathogens. It is clearly written and well-illustrated. I have only a few minor comments and questions.

**Response:** We thank the reviewer for appreciating our efforts and below provide the necessary explanations for the criticisms.

**Reviewer #2:**

Apte and colleagues investigate the functional significance of an RNA thermosensing (RNAT) element within the 5’ untranslated region (UTR) of the ciaR mRNA in Streptococcus pneumoniae (SPN). CiaR, a two-component system (TCS) response regulator (RR), is highly conserved in the streptococcus genus. Previous studies have established that CiaR controls phosphorylcholine (PCho) biogenesis and surface deposition in SPN via positive regulation of the lic operon. PCho is an important surface determinant of SPN adhesion and invasion: PCho interacts with host Platelet Activating Factor Receptor (PAFR), whose surface expression is highly on the respiratory epithelium and upregulated under inflammatory conditions. It follows that CiaR-dependent control of surface PCho may control SPN invasiveness. Here, the authors test the hypothesis that temperature-dependent control of ciaR mRNA translation dictates surface PCho levels which, in turn, controls the switch from colonization to invasive disease.

The authors have generated a good set of tools to probe their hypothesis. The manuscript and its figures are generally clear, but sometimes results are overstated.

The novelty of this work lies in the identification of the RNAT element in the 5’UTR of the ciaR mRNA . Whilst the findings presented convince that the 5’UTR of the ciaR mRNA is an RNAT element, the claim that it governs the commensal to pathogen switch is not substantiated by their in vivo infection data. Additionally, the discussion section reads more like a second introduction to the manuscript: there is a lack of interpretation of their results; explanation of mechanism, or any acknowledgement of limitations of the work. Some important experiments and a revision of the discussion section could be included to substantially strengthen the manuscript.

**Response:** We thank the reviewer for the constructive criticism and appreciating our findings. We have modified the discussion section and performed additional in-vitro and in-vivo experiments to answer all the criticisms.

**Reviewer #3:**

This manuscript describes the identification of an RNA termosensor affecting a characterized two component system (TCS) in the pneumococcus. Through a series of biophysical, cellular and in vivo experiments, the authors examine the functional consequences of the thermosensor and its effects on surface PCho and host:pathogen interactions.

The manuscript is well written, logically presented, and data support the conclusions.

**Response:** We thank the reviewer for appreciating our efforts.

**Part II – Major Issues: Key Experiments Required for Acceptance**

**Reviewer #1:** None

**Reviewer #2:**

**1.** The claim that the RNAT element governs the commensal to pathogen switch hangs on the data presented in Figure 6I. The authors reason that the increased abundance of the 5’UTRClosedciaRH strain compared to the WT and 5’UTROpenciaRH is due to the inability of the 5’UTRClosedciaRH strain to transition from commensal to virulent lifestyle. It follows that the authors are implying that the WT and 5’UTROpenciaRH strains are indeed transitioning from the nasopharynx, but they do not demonstrate that this is occurring. Could the authors perform an infection experiment that determines the fate of the three strains – i.e to show that they are, indeed, transitioning to invasive disease.

**Response:** We apologize for the clumsy wording in this section. Our intended meaning was that the two infection models together (i.e. the nasopharyngeal carriage model data and the pneumonia model data) suggested that the 5’UTRClosedciaRH strain retained an ability to establish an upper airway niche but showed reduced infectivity in higher temperature host tissues that are associated with disease. It is challenging to demonstrate a direct transition from carriage to disease in our colonization model, as it is a rare event (1-2% incidence for the D39 strain in the absence of other interventions). However, we do observe microaspiration of low bacterial numbers from nasopharynx to lungs in the colonization model and we have now compared those numbers for the open and closed strains at day 3 post infection (Suppl. Fig. 7). The data demonstrate lower bacterial numbers in the lower airway tissues for the closed variant (Suppl. Fig. 7A) and also a lower level of inflammatory cytokine production in both nasopharynx and lungs (Suppl. Fig. 7B,C). There are several possible interpretations for this finding, but these are not mutually exclusive:

1. Reduced numbers of lower airway bacteria leads to reduced inflammatory cytokine production.

2. Reduced expression of CiaRH-regulated virulence factors in the upper airway leads to reduced inflammatory cytokine production.

Inflammation drives much of the pathology associated with symptomatic pneumococcal lung infection, and both the above interpretations are consistent with the idea that a translation-permissive *ciaRH* UTR is required for establishment of inflammation-promoting infection in the lungs. We have updated the relevant section of the manuscript and removed the poorly worded sentence.

**2.** The authors have looked for the RNAT element in the 5’UTR of ciaR mRNA in 6 SPN serotypes, and from this, have concluded that “The heat responsive nature of CiaRH was found to be perfectly conserved across serotypes as well as in S. mitis, an evolutionary descendent of SPN” – Lines 631-2. To date, 107 serotypes have been identified and thousands of SPN genome sequences are available for analysis. From the data presented, it is clear that translation of ciaR mRNA is regulated by temperature in the serotype 2 strain D39. Can the authors do more extensive analysis of the conservation of the RNAT element, which would help the impact of the paper (i.e broaden its scope)? There is great diversity in the propensity for certain serotypes to colonize or cause invasive disease, and if the authors wish to claim that thermal regulation of ciaR translation is one of the most important factors that dictates this switch, better defining its conservation across serotypes would greatly reinforce this idea. Furthermore, CiaR is highly conserved in streptococcal species, again, for which sequences are in abundance. Conservation beyond S. mitis should be explored and discussed.

**Response:** We have now looked at the conservation of the 5’ UTR in a larger (>300) selection of SPN genomes, including isolates from 39 different serotypes. The region of the 5’ UTR with RNA-T structure (sequence: AATAAGGAGGAGTTTCTC) is 100% conserved across all genomes tested. We also performed a BLAST search to identify ciaR homologs in other bacterial species, retrieving >3000 genomes. Conservation of the 5’ UTR RNA-T element is high amongst closely related streptococcal species, with 100% conservation in *S. mitis* and four other streptococcal species and significant homology *in S. oralis, S. infantis, S. australis* and *S. nidrosiense* (16/18 base pairs). Full datasets can be found Supplementary Dataset 1, and we have updated Supp. Fig 3A to reflect the new analysis. The results are also described in the text (Page 8, Line 267-269; Page 8-9, Line 273-276) and corresponding methods is included (Page 17-18, Line 587-602).

**3.** As it stands, the discussion section reads like an expansion of the introduction and lacks interpretation of the authors results, explanation of mechanism, or acknowledgement of limitations of the work. Every paper has flaws. One example is the 5’UTRClosedciaRH strain. Although the approach of mutating the RNAT is an elegant one, the resulting 5’UTRClosedciaRH strain has roughly half the amount of protein than WT. Although this could be considered a small change, transcription factors (TF) are often present at low copy numbers per cell compared to other proteins; therefore, small changes in the amount of a TF can have far reaching consequences, particularly if the regulon is large. This is nicely illustrated in this work, where, for example, their 5’UTR mutants, which have ~1.4-fold/0.5 fold change in CiaR protein (open/closed, respectively), display statistically significant differences in surface PCho levels (Fig 3C/D/F/G). CiaR regulates a number of genes in SPN and the effect of the authors RNAT mutations are likely pleiotropic. It has been shown previously that a ciaR deletion mutant is attenuated in infection models, which can be relieved by overexpression of htrA. Have the authors considered how the likely reduced htrA expression in their 5’UTRClosedciaRH strain may confound interpretation of their results in Figure 6? Another example is the finding that ciaR transcripts do not change with temperature. This conflicts with previous studies looking at the effect of temperature on the transcriptome of SPN (see below). This is another example of an important point that should be discussed.

**Response:** We thank the reviewer for this comment. As suggested, have now completely remodeled the discussion to a more succinct version, highlighting the possible limitations and future scope of the study. We have also modified our interpretation of the in vivo data, to acknowledge that virulence changes might be attributable to CiaRH-regulated virulence factors beyond PCho. With regards to the study of Gazioglu and colleagues, where they observed that *htrA* overexpression relieved the effects of *ciaRH* deletion, it was notable that this effect was only seen at 40ºC, suggesting that HtrA might play an important role in CiaRH-mediated temperature responses (Page 14, Line 463-466; 472-497).

**Reviewer #3:**

**1.** The CiaR RNAT appears to be composed by base pairing of four pairs of nucleotides, which is a rather minimal structure compared with the S mitis and Nm RNATs presented here and with other thermosensors. Of note, the S mitis RNA thermosensor includes more 5' sequence, while the Spn ciaR 5' UTR is short in comparison. I cannot find data on mapping of the 5' UTR of the ciaR mRNA in the literature or the paper; if this is not available, the authors should confirm the TSS experimentally. This will provide more confidence that their predicted structure represents what happens in vivo.

**Response:** We thank the reviewer for pointing this out and have now cited the article which has experimentally annotated the *ciaR* transcriptional start site in *Streptococcus pneumoniae* D39 (reference # 39). The same can be viewed in the online tool Pneumobrowse developed by Veening lab

(https://pneumobrowse2.veeninglab.com/?session=share-0jvMD-xdy6&password=aV15u).

**2.** The open and closed ciaR RNATs have been incompletely characterized. Analysis using a GFP reporter or in vitro translation should be performed.

**Response:** As suggested by the reviewer, we have cloned the engineered 5’UTRs (Open and Closed forms) in front of *gfp* and analysed the GFP protein expression at different temperatures. Our results show that although there is no variation in GFP levels across temperatures within a single strain, GFP levels differ significantly in between the reporter strains with open and closed ciaR RNATs. This result is shown in Suppl. Fig. 4A and mentioned in the results section (Page 9-10, line 311-315) and methods (Page 18, line 614-618).

**3.** Findings with in vitro transcription/translation do not distinguish between the effects of these processes. The authors can simply decouple them by generating mRNA in vitro, then adding fixed amount of transcript to transcription/translation assays to discriminate between effects due to transcription, or as proposed, translation initiation.

**Response:** As suggested by the reviewer, we decoupled the in vitro transcription-translation reaction. We first generated RNA by in vitro transcription using T3 RNA polymerase and then used equal amounts of RNA for in vitro translation. Our result corroborates with our earlier findings and suggest translation-based regulation of *ciaR* RNAT (Suppl. Fig. 1F). This is described in the results (Page 8, Line 246-249) and methods (Page 20, Line 691-697).

**Part III – Minor Issues: Editorial and Data Presentation Modifications**

**Reviewer #1:**

**1.** Pcho levels are elevated in the lungs of infected mice (Fig. 1A), presumably reflecting a physiological body temperature of 37°C. However, most of the experiments in the manuscript were conducted at 33 and 39°C. This raises the question of whether a normal mammalian body temperature (37°C) is sufficient to trigger the CiaR-mediated response, or if an elevated, febrile temperature is necessary. A melting temperature of 34°C suggests that 37°C should elicit a substantial response.

**Response:** As suggested, we evaluated CiaR expression pattern at 33°C (nasopharyngeal temp.), 37°C (body tem.) and 39°C (febrile temp.) and found that CiaR levels increased only at 39°C. However, the *ciaR* RNA melting temperature was determined to be 34°C in *in vitro* analyses. This discrepancy could be attributable to the *in vitro* conditions employed in the melting temperature assay, or assay sensitivity limitations of our subsequent *in vitro* and *in vivo* conditions. The data are shown in Suppl. Fig. 1D-E, and described in the text (Page 7, Line 225-227).

**2.** Fig. 2D: The RNA-T upstream of ciaR possesses several intriguing features that merit some discussion, e.g. in the context of discussing Fig. 6. With a hairpin of only four base pairs, it is unusually short, yet remarkably efficient (Fig. 6 should reflect this unusual feature instead of showing nine base pairs). Additionally, it exhibits an uncommon structural arrangement: The sequence that base-pairs with the SD sequence is located downstream of it. In most cases, the base-pairing nucleotides are positioned upstream of the SD sequence. Here also, Fig. 6 should be corrected by positioning the ribosome to the left side of the hairpin.

**Response:** We appreciate the detailed observations of the uniqueness of ciaR RNAT structure. As suggested, the diagram has been modified with respect to ribosome positioning on the hairpin. We have also now discussed the short nature of the RNAT in the discussion section (Page 13, Line 450-454).

**3.** Fig. 3A: What was the rationale for constructing a “closed” variant with six nucleotide substitutions, including four mutations in the tetraloop? The predicted structure of this variant introduces a bulge within the SD sequence, which could potentially reduce the overall hairpin stability. At first glance, a single mutation (T15C, or better U15C, because the sequence is RNA) might have been sufficient to stabilize the hairpin.

**Response:** We agree with the reviewer’s point that incorporation of a single mutation would have possibly created a closed *ciaR* RNAT version. However, in-silico prediction using mFold suggested that a single mutation of T15C is not sufficient to completely close the loop. Instead we chose to close the RBS region fully by multiple substitution for creation of the 5’UTR-CiaRClosed strain. To our satisfaction, this strain restricted RNAT opening even with the small bulge in between.

**4.** Fig. 3D: “ciaR levels” on the Y-axis should be “CiaR (= protein) levels”.

**Response:** We have changed the Y-axis label to “CiaR levels”.

**5.** “Expression” is typically defined as the process by which information from a gene is used in the synthesis of a functional gene product, a protein or non-coding RNA. The authors often use “expression” in a sense that is not compatible with this definition, e.g. labeling of the Y-axis in Fig. 1D “Pcho/Enolase expression” needs to be changed to “Pcho/Enolase levels”. The same applies to Fig. 2B and F. Another example: “Pcho expression2 in lines 167 and 177. Please check the text carefully.

**Response:** We agree with the reviewer and have made the required changes in Fig. 1D, 2B 2F and in text (Page 6, Line 167 and 178).

**6.** Line 560: V. cholerae.

**Response:** This has been removed as we completely changed the discussion section.

**7.** Lines 631 and 636: Statements like the “heat responsive nature of CiaRH” or “thermosensing activities of CiaRH” are not correct because the mRNAs rather than the proteins respond to temperature.

**Response:** We agree and have made the necessary changes.

**Reviewer #2:**

**1.** Can the graphs in Figure 1 A and B be combined to facilitate comparisons between the two temperatures? I understand that for this to occur, the data will have to be presented in the same way as A. If you think the paired data are more important and do not wish to combine the graphs, then I do not think comparisons should be made between the two sets of data, as they are in the text (lines 172-174).

**Response:** We have added the 40ºC exposure nasopharynx data to Figure 1A. There is a clear trend towards increased PCho in the 40ºC group, but the difference in not significant in ANOVA analysis (P = 0.09). We have updated our description of the results to reflect this (Page 6, Line 174-175).

**2.** The focus of the manuscript is the RNAT element, but CiaR is a TCS RR which is also subject to regulation by its cognate histidine kinase, CiaH. Although the signal(s) that CiaH senses directly are not known, this layer in the regulation of CiaR activity (following translation) is ignored. This is an aspect of the mechanism that could be discussed in the discussion. Should we expect higher CiaR protein levels to lead to greater activation/repression of the promoters it regulates in the absence of CiaH’s signal? Could the authors expand on what is known about CiaRH signaling in the introduction to facilitate a wider audience gaining a greater mechanistic understanding of their findings?

**Response:** We thank the reviewer for this insightful comment. We have now included additional information on the CiaRH operon and its activation in the discussion. Since not much is known about the ligands of CiaH, we believe that further study is necessary to understand how another signal in addition to temperature would affect the CiaR activity. We have discussed this point and the potential requirement for thermoregulation to mediate its effects only in the presence of an additional direct signal via CiaH (Page 14, Line 472-497).

**3.** Some of the language used is overly strong, or not appropriate, and does not always reflect the data:

**a.** Line 1: “Commensal to pathogen switch in Streptococcus pneumoniae is governed by a thermosensing master regulator” – influenced would be more appropriate.

**Response:** As suggested, we have changed the title of the manuscript.

**b.** Line 160: “Temperature shift is the cue for the commensal to invasive switch for S. pneumoniae”. The data that follows supports that temperature is a cue, but does not show that it is the cue.

**Response:** We modified this in the revised version as recommended (Page 6, Line 160).

**c.** Line 177: The key factor is not being determined here – only 3 factors are being tested (temperature, ATP, and TNF). You are asking if increased temperature is a key factor, or rather, if changes in temperature are sufficient to drive changes in PCho expression.

**Response:** As suggested, we made the necessary changes in the sentence (Page 6, Line 178).

**4.** Additional notes on the text and figures:

a. Figure 1C: Can the authors comment on the bands at 15 kDa in the western blot and why they are absent in the temperature samples? Were all the blots performed separately, as presented? This would not allow for reliable quantification.

**Response:** We have performed additional experiments and replaced the temperature part of the immunoblot in Fig. 1C. The current blot has prominent 15kDa bands in temperature samples. However, the reviewer pointed out correctly that the immunoblots for temperatures and ATP/TNF-α were run separately. The normalizations were done for each individual sample against respective enolase levels. Post normalization, fold changes were calculated using mock treatment as a control for the TNFα / ATP blot and 33ºC for the temp blot. To alleviate any confusions, we have separated the graphs and mentioned this in the figure legend (Page 24, Line 818-825).

**b.** Figure 2C: Can the authors please comment on their finding that ciaR transcripts do not change with temperature, which conflicts with a previous study that used a microarray to investigate changes in the transcriptome at different temperatures: Gazioglu O, Kareem BO, Afzal M, Shafeeq S, Kuipers OP, et al. Glutamate Dehydrogenase (GdhA) of Streptococcus pneumoniae is required for high temperature adaptation. Infect Immun 2021;89:e0040021.

**Response:** Gazioglu et al., performed DNA microarray analysis to reveal the differences in gene expressions when *S. pneumoniae* was grown at different temperatures. Among other genes, the authors found that *ciaR* transcripts were down-regulated. However, differential expression of ciaR obtained by microarray has not been validated by gene specific analysis. Our findings with *ciaR* specific qRT-PCR primers strongly suggest that transcript levels are unchanged at higher temperatures. This experiment was performed with 8-10 replicates, giving us confidence in the findings. Also, CiaR controlled genes, such as *htrA*, are reported to increase at higher temperature, which supports our results over the findings described in the studies by Gazioglu et al.

**c.** Line 115: SPN has 13 TCSs, not dozens.

**Response:** As pointed out, this has been changed to indicate a precise number of TCS (Page 4, Line 115).

**d.** Lines 139-142: Can the authors change the emphasis from nasal dwelling to the respiratory tract. Pseuomonas aeruginosa and Acinetobacter spp., can be found colonizing the nasal cavity in certain circumstances, they are not true nasopharyngeal commensals, like N. meningitidis and H. influenzae.

**Response:** As per the suggestion, we have made the necessary change (Page 5, Line 138-141).

**e.** Line 196: “Pneumococcal expression of phosphorylcholine and consequent invasiveness in in-vivo and in-vitro is triggered by a temperature shift.” In vivo invasiveness is not tested here.

**Response:** We agree with the reviewer and have changed the title accordingly (Page 24, Line 807-808).

**Reviewer #3:**

**1.** A key aspect of this work is linking the effects of temperature on surface expression of PhoC and the CiaRH TCS. For this, data in Supplementary Fig 2 are critical, and I suggest incorporating this in the text of the main manuscript.

**Response:** We thank the reviewer for carefully looking at the data, including the supplemental figures. The data shown in Suppl. Fig. 2 primarily depicts that ciaRH deletion reduces PCho expression and impedes pneumococcal invasion. We created the ciaRH knock-out mutant and performed these set of experiments to re-establish the key role of the CiaRH system in SPN lifestyle, but these phenotypes have already been described in previous studies. Furthermore, these experiments do not address how subtle changes in CiaR expression could trigger changes in surface moieties in SPN. We addressed this issue via the RNAT system that we deciphered. For these reasons, we believe that data pertaining to ciaRH deletion mutant is best described as supplementary information.

**2.** The discussion is very broad in most part, and more should be devoted to critical appraisal of work presented in the manuscript, than the rather general review of regulation by pathogens in response to host signals that currently occupies a lot of the discussion. What else does this TCS control in Spn? What are the strengths/limitations of the study? Could the effect of the closed ciaR promoter (which reduces translation irrespective of temperatue) in vivo be mediated by effects on other genes regulated by the CiaRH TCS? How can the authors differentiate between this and effect on PhoC; the effect on PhoC is the major focus of the cellular studies and explored in vivo by the addition of exogenous PhoC?

**Response:** Thank you for this comment, which aligns with suggestions from reviewer 2. As noted in our response to reviewer 2, we have now reworked the discussion to acknowledge the potentially vital contributions from other (non-PCho) members of the CiaRH regulon. We have also defined the CiaRH regulon more fully.

**3.** Line 49 and elsewhere: umbrella system. I dont like this. why not just delete act as umbrella systems, and just say dictate pleiotropic phenotypes?

**Response:** As suggested, necessary changes have been made (Page 2, Line 45; Page 4, Line 106).

**4.** Line 65 The nasopharynx?

**Response:** As suggested, we replaced “Our nasopharyngeal area” with “The nasopharynx” (Page 3, Line 65).

**5.** Line 88 the underpinning.?

**Response:** As suggested, we added “the” before “underpinning” (Page 4, Line 88).

**6.** Fig 1B How can you be sure these results are from Spn as the samples appear to be just washes?

**Response:** Washes were collected in PBS containing gentamicin, to kill any non-SPN bacteria. After flow cytometry data collection, remaining wash was plated onto gentamicin blood agar to confirm presence of pneumococci and absence of other organisms. This is mentioned in the methods section (Page 17, Line 559-561).

**7.** Line 220 You cannot use SEM with such a sample size. Please do more replicates if you want to calculate SEM.

**Response:** The analysis was done based on Standard deviation (SD) and not SEM. We have corrected this in the manuscript (Page 24, Line 835).

**8.** Line 247 This is looking at protein levels and not the levels of translation per se.

**Response:** As suggested we have changed “examined the transcriptional and translational levels” to “examined the transcript and protein levels” (Page 7, Line 221).

**9.** Line 255 what is the predicted Tm of this structure? There is little base pairing, and could unfold at temperatures lower than are relevant for a bacterium at or around 37ºC.

**Response:** The predicted Tm of the *ciaR* RNAT using Qiagen’s prediction tool (https://geneglobe.qiagen.com/us/tools/tm-prediction) suggests it to be 48ºC. However, our experiments suggest that the *ciaR* RNAT opens up for translation at ~39ºC. This could be due the presence of different salts and concentration of the RNA in the bacterial cytoplasm.

**10.** Line 280 It is unusual for the interaction between mRNA and ribosomes to be measured by Kd given it is a multistep complex interaction... usually delta G is employed. Also affinities are extremely high.

**Response:** We agree with the reviewer’s point that generally ∆G value is suggestive of the interaction between RNA and protein and we appreciate that ribosome binding to RNA is a multistep process. However, the results we obtained using the Monolith system with lesser sample volumes encompassing a small RBS region were very promising. The same ribosomal solution showed no binding at all with the control RNA used, which lacked the RBS region, strengthening our confidence in the microscale thermophoresis (MST) data. The system quantified the interaction as KD values and so this is how we represented them in the manuscript.

**11.** 362 Similar to the wt RNAT, what is the predicted Tm. These changes seem to have very little effect on the predicted structure (esp for close, although these are all GC pairs).

**Response:** We predicted Tm of WT, open and closed *ciaR* mRNA sequences using Qiagen’s online tool (https://geneglobe.qiagen.com/us/tools/tm-prediction). The platform suggested a Tm of 48ºC for WT *ciaR* RNAT sequence, 38ºC for the open sequence and 56ºC for the closed sequence. These predicted Tm are higher than the temperature range within which we observed reproducible differences in mRNA translation between sequences. We attribute these differences to the presence of different salts and differences in the concentrations of RNA in our experimental systems vs those used by the predictive tool.

**12.** 448 Please show data for 4A in supplementary data

**Response:** As suggested, we have moved Fig. 4A to Suppl. Fig. 5G.
